# Thyroid cancer incidence trend and association with obesity, physical activity in the United States

**DOI:** 10.1186/s12889-022-13727-3

**Published:** 2022-07-12

**Authors:** Biaoyou Chen, Zhaomin Xie, Xuwei Duan

**Affiliations:** 1 Department of Head and Neck Surgery, Guangxi Medical University Cancer Hospital, 71 Hedi Road, Nanning, 530021 China; 2 Department of Medical Oncology, Cancer Hospital of Shantou University Medical College, 22 Xinling Road, Shantou, 515031 China

**Keywords:** Thyroid cancer, Incidence, Obesity, Physical activity

## Abstract

**Background:**

State-level racial/ethnic and age differences and the temporal trend of thyroid cancer (TC) incidence in the USA remain unknown. Our research purposes include: Characterizing state-level temporal variation in TC incidence; examining the disparities of TC incidence by state-level race/ethnicity and age; performing an ecological correlation between TC incidence and obesity/physical activity.

**Methods:**

TC incidence data during 2000–2017 were extracted from the United States cancer statistics. Using joinpoint regression to evaluate TC incidence trends. Annual percent change (APC), average APC (AAPC) and incidence rates were calculated. The obesity prevalence and physical activity level at the state-level were extracted from Behavioral Risk Factor Surveillance System, and the association between state-level AAPC of TC and obesity/physical activity was tested by Pearson correlation coefficient.

**Results:**

We found that the TC incidence had shown an overall downward trend in recent years, but 10 states continued increasing. There were significant differences in state-level race/ethnicity (non-Hispanic Whites as a reference) and age group (45–59 age group as a reference) incidence: Incidence Rate Ratio (IRR) was 0.4–1.2 for non-Hispanic Blacks, 0.7–1.6 for non-Hispanic Asian and Pacific Islanders, 0.4–1.2 for non-Hispanic American Indians/Alaskan Natives, and 0.5–1.3 for Hispanics. High IRR in young people were distributed in northern USA, while in older people were distributed in south. The state-level obesity/physical activity level and AAPC had a weak correlation (*r* = 0.34, *P* = 0.016) and inverse weak correlation (*r* = -0.29, *P* = 0.037), respectively. The AAPC of states with a consistent increasing trend had an extremely strong correlation with obesity prevalence (*r* = 0.80, *p* = 0.006), and an inverse strong correlation with physical activity level (*r* = -0.65, *P* = 0.04).

**Conclusions:**

Thyroid cancer incidence in 10 states continued increasing. State-level variation in race/ethnicity and age group incidence were found. Lifestyle and environmental factors may interfere with the incidence trend of TC in the USA.

**Supplementary Information:**

The online version contains supplementary material available at 10.1186/s12889-022-13727-3.

## Introduction

The incidence of thyroid cancer (TC) is increasing, and this disease is expected to become the fourth leading type of cancer in the worldwide [1]. In the USA, the incidence of TC had increased rapidly over the past decades, from 4.56 cases per 100,000 person-years in the mid-1970s to 14.42 cases in the early 2010s [2]. But the rise of TC incidence rates slowed down in the USA after 2009 [3]. Lee et al. noticed that after 30 years of exponential growth, the incidence of TC in the USA began to decline by a statistically significant rate for the first time from 2015 to 2017[4], the decline mainly owing to the tumors ≤ 1 cm, and the tumors > 1 cm did not find a significant decline.

Due to the lack of the latest relevant study, it is not clear whether the overall decline trend is consistent in all states, race/ethnicity, and age groups. Few state-level trends in TC incidence have been reported in the USA, previous studies were mainly carried out at the national level [5, 6]. TC incidence had geographical distribution disparity in the USA [7, 8]. Various environmental factors and biological factors [9, 10], diet [8] may have an impact on state-level TC incidence. In addition, the disparity in the incidence of TC among various race/ethnicity is obvious. It is generally believed that Whites and Asian and Pacific Islanders had the highest incidence, while Blacks had the lowest incidence. Age is usually considered to be associated with the TC incidence [11]. It had been reported that the incidence of TC in adolescents had continued to increase in recent years [12]. Kotwal et al. believed that TC incidence trend deceleration mainly occurred in non-Hispanic Whites and elderly, but not in young, Hispanic and Black subgroups [13]. In addition, Weeks et al. found that from 2007 to 2014, the incidence trend of TC in patients aged 15 to 54 years old decreased significantly in the order of white, Asian, Hispanic, Native American and African American. For patients aged 55 to 84 years old, African Americans maintained significantly lower rates than all other race/ ethnicity groups. For patients aged ≥ 85 years old, there were no statistically significant differences in TC incidence rates among race/ethnicity [10]. Focus on trends in incidence of TC at the state-level and racial/ethnic, age levels may help health care departments to develop management schemes.

According to the previous studies, the increases in the incidence rate of TC in the USA were found both in localized tumors and advanced stage TC [4, 14]. Some studies have attributed the rising TC incidence mainly to more intensive surveillance and improved diagnostics [15]. Furthermore, lifestyles and environmental factors may be also at play [15, 16]. The metabolic burden of the American population continued to increase, and obesity is considered to be one of the causes of the TC [17]. In addition, lack of exercise is thought to be one of the causes of many malignant tumors [18]. There is a disparity in obesity prevalence and physical activity level among states in the USA. The state-level obesity/physical activity level and TC incidence trends may have an ecological correlation. Ecological correlation analysis can help us identify the causes of trend variation. Apart from that, environmental factors are also considered to be an important part of intervention in the TC incidence trends [5, 10, 12]. Lee et al. also indicated the first significant decline in the TC incidence, which cannot exclude the role of environmental factors [4]. Environmental factors such as ionizing radiation, water pollution, artificial chemicals, climate factors and volcanic eruption may be related to TC [19–22].

In summary, the overall incidence of TC in the USA has been declining, but the latest incidence trend in some groups and regions is still unclear. This study will focus on the following aspects: describing the incidence trend of TC at the state level; examining the state-level ethnic/racial and age disparity of TC incidence in the USA during 2000–2017; and further exploring the related factors affecting the incidence trend.

## Materials and methods

### Data source

The United States Cancer statistics (USCS) is the official federal most comprehensive cancer incidence statistics center. Each of the cancer registries of the USCS capture more than 90% of all cases; and more than 97% of cases pass computerized validity and logic checks [23]. These statistics include cancer registry data from CDC’s National Program of Cancer Registries (NPCR) and the National Cancer Institute’s (NCI) Surveillance, Epidemiology, and End Results (SEER) Program. Through NPCR, CDC supports central cancer registries in 46 states, the District of Columbia, Puerto Rico, the U.S. Pacific Island Jurisdictions, and the U.S. Virgin Islands. SEER collects and publishes cancer incidence and survival data from population-based cancer registries in 19 U.S. geographic areas, including 5 states [24]. Cancer surveillance data from CDC and NCI are combined to become USCS [25]. To know the TC incidence trends, we analyzed the data from the USCS (the CDC Wide-ranging Online Data for Epidemiologic Research platform) between 2000 and 2017.

Behavioral Risk Factor Surveillance System (BRFSS) is a national monitoring system, which mainly collect prevalence data among US residents regarding their risk behaviors and preventive health practices that can affect their health status through telephone survey. State-level obesity prevalence and physical activity level were obtained for all 50 states and DC, and stratified by sex and race/ethnicity. The obesity variable we defined as follows: BMI 30.0–99.8 kg/m2; and the physical activity variable was defined as individuals who participated in 150 min or more of aerobic physical activity per week [26].

### Definition of variables

The variables we obtained from USCS. Race/ethnicity includes Hispanic, non-Hispanic White (White), non-Hispanic Black (Black), non-Hispanic Asian and Pacific Islander (API), and non-Hispanic American Indian/Alaskan Native (AI/AN). Age groups include < 30 years, 30–44 years, 45–59 years, 60–74 years, and ≥ 75 years. Sex includes female and male. States include 50 states and District of Columbia in the USA.

The research data in this study are from the USCS database. Personal information will not be disclosed, so ethical review should be exempted.

### Statistical analysis

Population-based age-adjusted incidence rates and 95% Confidence Interval (CI) were calculated for the years using the 2000 US standard population reported per 100,000 person-years. The Joinpoint Regression Program software (version 4.9.0.0) was used to estimate the trends of TC incidence. This version of the Joinpoint Program did not provide an exact *p* value for AAPC, only *p* < 0.05 or *p* > 0.05 was provided. This software used the best-fitting log-linear regression model to identify the calendar years when APCs changed significantly, and Monte Carlo Permutation method was applied to evaluate the tests whether or not show a statistically significant change in trend. The program selects joinpoints that starts with the minimum number of joinpoint (i.e. no joinpoints), and examined whether more joinpoints are statistically significant [27].

The temporal trend of TC incidence was quantified by overall and sex, age, race/ethnicity, states and subtypes. The main evaluation indicators are Annual Percent Change (APC), Average APC (AAPC) and 95% CI. Incidence Rate Ratio (IRR) was calculated to quantify state-level racial/ethnic and age variations in TC incidence. In addition, the heat maps to show the state-level IRRs were generated by R software (4.0.4). Pearson correlation analysis was used to analyze the ecological correlation between state-level AAPC and obesity/physical activity level. Two-sided *P* < *0.05* was considered statistically significant.

## Results

### Age-adjusted incidence and trends of TC incidence in the study period

During 2000–2017, the USCS registry collected a total of 683,157 TC cases with an overall incidence rate of 12.1 cases/100,000 person years (PY). The incidence rate reflects large gender differences, with an incidence of 17.9 cases/100000 PY for female and 6.2 cases/100000 PY for male. The TC incidence for API (13.2 cases/100,000 PY) was the highest in all race/ethnicity, followed by Whites (12.9 cases/100,000 PY) and Hispanics (11.4 cases/100,000 PY), AIs/ANs (9.0 cases/100,000 PY) and the lowest among Blacks (7.8 cases/100,000 PY). The highest TC incidence rate was found in 60–74 age group (22.3 cases/100,000 PY), followed by 45–59 age group (20.8 cases/100,000 PY), 30–44 age group (16.3 cases/100,000 PY), ≥ 75 age group (15.5 cases/100,000 PY), and the lowest incidence was found in < 30 age group (2.9 cases/100,000 PY) (As shown in Table 1).

**Table 1:** Trends in Age-Adjusted Thyroid Cancer Incidence Rates by Demographic factors, 2000–2017

	2000–2017		Incidence Rate cases/100000 PY				Trend 1/ Trend 3			Trend 2/ Trend 4				2000–2017	
Demographic factors	No of new cases	Age adjusted/ 100 000 (95% CI)	2000	2009	2015	2017	Years	APC (95%CI)	*P* Value	Years	APC (95%CI)	*P* Value		AAPC (95%CI)	*P* Value
Overall	683,157	12.1(12.1,12.1)	7.4	13.6	14.7	13.2	2000–2009	7.3^a^ (6.8, 7.7)	< 0.001	2009–2015	1.6^a^ (0.9, 2.3)	< 0.001		3.6^a^ (3.2, 4.1)	< 0.05
							2015–2017	-5.8^a^ (-8.7, -2.8)	0.002						
Sex															
Female	514,414	17.9(17.8,17.9)	10.9	20.1	21.8	19.4	2000–2009	7.2^a^ (7.0, 7.5)	< 0.001	2009–2012	2.3^a^ (0.6, 4.2)	0.017		3.6^a^ (3.2, 4.0)	< 0.05
							2012–2015	0.5 (-1.1, 2.2)	0.468	2015–2017	-5.6^a^ (-7.3, -3.9)	< 0.001			
Male	168,743	6.2(6.1,6.2)	3.9	6.9	7.4	6.9	2000–2009	6.9^a^ (6.2, 7.7)	< 0.001	2009–2014	2.0^a^ (0.4, 3.7)	0.021		3.8^a^ (3.1, 4.5)	< 0.05
							2014–2017	-2.3 (-4.8, 0.2)	0.068						
Race/ethnicity															
AI/AN	3719	9.0 (8.7,9.3)	4.8	9.6	11.2	10.8	2000–2013	7.3^a^ (6.1, 8.6)	< 0.001	2013–2017	-1.7 (-6.7, 3.6)	0.493		5.1^a^ (3.7, 6.6)	< 0.05
API	37,351	13.2 (13.0,13.3)	8.6	14.1	15.9	14.3	2000–2003	2.3^a^ (0.4, 4.3)	0.025	2003–2009	7.4^a^ (6.7, 8.1)	< 0.001		3.1^a^ (2.7, 3.5)	< 0.05
							2009–2014	2.8^a^ (2.1–3.5)	< 0.001	2014–2017	-3.9^a^ (-4.9, -2.8)	< 0.001			
Blacks	49,909	7.8 (7.7,7.9)	4.9	8.5	9.6	8.1	2000–2013	5.8^a^ (5.2, 6.5)	< 0.001	2013–2017	-5.4^a^ (-8.0, -2.6)	0.001		3.1^a^ (2.3, 3.9)	< 0.05
Whites	506,759	12.9 (12.9,13.0)	7.9	14.8	15.8	14.2	2000–2009	7.5^a^ (7.1, 8.0)	< 0.001	2009–2015	1.3^a^ (0.5, 2.1)	0.003		3.7^a^ (3.2–4.2)	< 0.05
							2015–2017	-5.7a (-8.9, -2.3)	0.004						
Hispanic	77,526	11.4 (11.3,11,5)	7.0	12.1	13.8	12.9	2000–2009	6.3^a^ (5.5, 7.1)	< 0.001	2009–2015	2.5* (1.1, 3.8)	0.002		3.7^a^ (2.9–4.6)	< 0.05
							2015–2017	-3.4 (-8.7, 2.2)	0.199						
Age (years)															
< 30	68,650	2.9 (2.9–2.9)	2.1	3.0	3.5	3.3	2000–2015	3.8^a^ (3.5, 4.2)	< 0.001	2015–2017	-5.5 (-12.4, 1.9)	0.128	2.7^a^ (1.8, 3.6)		< 0.05
30–44	183,261	16.3 (16.3,16.4)	10.3	17.3	20.8	18.7	2000–2006	6.1^a^ (5.0, 7.2)	< 0.001	2006–2009	8.3^a^ (3.0, 14.0)	0.007	3.6^a^ (2.6, 4.6)		< 0.05
							2009–2015	2.0^a^ (0.9, 3.0)	0.003	2015–2017	-5.3^a^ (-9.5, -0.8)	0.027			
45–59	229,817	20.8 (20.7–20.9)	12.3	21.8	25.2	22.7	2000–2009	7.5^a^ (7.1, 8.0)	< 0.001	2009–2014	2.0^a^ (0.9, 3.1)	0.002	3.8^a^ (3.4, 4.3)		< 0.05
							2014–2017	-3.6^a^ (-5.3, -1.9)	0.001						
60–74	150,976	22.3 (22.2–22.5)	12.4	25.6	25.8	23.0	2000–2008	9.4^a^ (8.5, 10.2)	< 0.001	2008–2013	2.1^a^ (0.4, 3.8)	0.018	4.1^a^ (3.4, 4.7)		< 0.05
							2013–2017	-3.5^a^ (-5.0, 2.0)	< 0.001						
≥ 75	50,453	15.5 (15.3–15.6)	10.1	17.8	17.3	15.7	2000–2008	7.7^a^ 6.8,8.5)	< 0.001	2008–2014	0.8^a^ (-0.5,2.2)	0.210	3.0^a^ (2.2,3.7)		< 0.05
							2014–2017	-4.7^a^ (-7.6,-1.7)	0.006						

^a^The difference was statistically significant

National TC incidence increased with an annual rate of 3.6% (AAPC) from 2000 to 2017 (*P* < *0.05*). During the study period, joinpoint regression identified two inflection points (2009 and 2015), and generated three linear segments (2000 to 2009, 2009 to 2015 and 2015 to 2017). During 2000–2009, the incidence rate increased rapidly at 7.3% (*P* < *0.001*) per year, increased slowly to 1.6% (*P* < *0.001*) per year during 2009–2015, decreased significantly to 5.8% (*P* = *0.002*) per year from 2015 to 2017. TC Incidence rate for male increased at 6.9% per year (*P* < 0.001), for female 7.2% per year from 2000 to 2009 (*P* < 0.001). Subsequently, the incidence of TC in both male and female had reversed since 2009. The TC incidence rate of AIs/ANs increased with the highest AAPC of 5.1% per year (*P* < 0.05) among all race/ethnicity in 2000–2017, followed by Whites and Hispanics, both with an AAPC of 3.7% (*P* < 0.05) per year, Blacks and APIs with an AAPC 3.1% (*P* < 0.05) per year. After about 10 years of rapid growth, the incidence of TC in other racial/ethnic groups except for Hispanics and AIs/ANs has declined statistically in recent years. During the whole study period, the TC incidence rate among the 60–74 age group increased with the highest AAPC of 4.1% (*P* < 0.05) per year, < 30 age group with the lowest AAPC of 2.7% (*P* < 0.05) per year. More recently, the TC incidence for most age groups showed a descending trend, but there was no significant difference in the < 30 age group. More detailed information on incidence trends was shown in Table 1.

### Changes in state-level TC incidence over the study period

State-level age-adjusted TC incidence, changing trends and AAPCs during the study period were shown in Table 2. During the study period, the lowest TC incidence in all states was in Alabama (8.2 cases/100000 PY), the highest was in Pennsylvania (17.1 cases/100000 PY), and the IRR between the highest and lowest states was 2.1. In 2000, the lowest incidence was in Maine (6.9 cases/100000 PY), the highest was in New Jersey (16.7 cases/100000 PY), and the IRR between the highest and lowest incidence of states was 2.4. During 2000–2017, the incidence was increasing in almost all states. The states with the largest increase were in Oklahoma (AAPC, 8.1%/yr, *P* < *0.05*), Kentucky (AAPC, 6.1%/yr, *P* < *0.05*), Arkansas (AAPC, 6.1%/yr, *P* < *0.05*), and New Hampshire (AAPC, 6.1%/yr, *P* < *0.05*). Joinpoint regression analysis showed that the incidence continued to increase in 10 states, plateaued in 29 states, and decreased in 11 states in recent years (Table 2). From this point, only about 1/5 of the states have occurred a significant decline in TC incidence. In addition, the disparity in the incidence of state-level continued to decrease. In 2000, 2009 and 2015, the corresponding IRR between the highest and lowest incidence of states were 2.4, 2.3 and 1.9, respectively. However, the IRR decreased to only 1.8 in 2017.

**Table 2:** Trends in Age-Adjusted Thyroid Cancer Incidence Rates by States, 2000–2017

	2000–2017		Incidence Rate cases/100000 PY				Trend 1/Trend 3			Trend 2/Trend 4			2000–2017	
States	No of new cases	Age adjusted/ 100 000 (95% CI)	2000	2009	2015	2017	Years	APC (95%CI)	*P* Value	Years	APC (95%CI)	*P* Value	AAPC (95%CI)	*P* Value
**1. Rising trends**														
Arkansas	4578	9.0 (8.7, 9.2)		16.8	21.6	19.6	2001–2008	10.6^a^ (6.6, 14.7)	< 0.001	2008–2017	2.4^a^ (0.6, 4.3)	0.015	5.9a (4.1, 7.7)	< 0.05
Delaware	2189	13.2 (12.6,13.8)	11.2	19.4	21.4	19.6	2000–2017	2.6^a^ (1.1, 4.2)	0.002				2.6^a^ (1.1, 4.2)	< 0.05
Georgia	17,137	10.1 (9.9,10.2)	12.2	19.2	22.0	20.8	2000–2008	7.3^a^ (4.5, 10.2)	< 0.001	2008–2017	2.1^a^ (0.6, 3.7)	0.011	4.5^a^ (3.1, 5.9)	< 0.05
Iowa	6729	12.1 (11.8,12.4)	12.0	23.4	24.1	19.9	2000–2017	3.7^a^ (2.3, 5.1)	< 0.001				3.7^a^ (2.3, 5.1)	< 0.05
New Mexico	4977	13.6 (13.2,14.0)	10.1	24.5	27.5	24.7	2000–2017	2.2^a^ (1.0, 3.5)	0.002				2.2^a^ (1.0, 3.5)	< 0.05
North Dakota	1598	12.9 (12.3,13.5)		23.4	26.8	26.6	2000–2017	4.9^a^ (2.4, 7.6)	0.001				4.9^a^ (2.4, 7.6)	< 0.05
Oklahoma	6479	9.6 (9.3, 9.8)	7.1	17.9	23.8	20.8	2000–2006	16.9^a^ (7.4, 27.3)	0.002	2006–2017	3.5^a^ (1.3, 5.8)	0.004	8.1^a^ (4.9, 11.4)	< 0.05
South Dakota	1589	11.5 (10.9,12.0)		22.7	22.3	19.0	2001–2017	3.9^a^ (1.7, 6.1)	0.002				3.9^a^ (1.7, 6.1)	< 0.05
Vermont	1443	12.1 (11.5,12.8)		22.9	20.8	18.9	2002–2017	2.2^a^ (0.1, 4.3)	0.044				2.2^a^ (0.1, 4.3)	< 0.05
Wisconsin	11,610	11.1 (10.9,11.3)	10.0	19.7	21.7	21.2	2000–2009	7.8^a^ (5.5, 10.1)	< 0.001	2009–2017	2.1^a^ (0.3, 3.9)	0.028	5.0^a^ (3.7, 6.4)	< 0.05
**2. No significant change**														
Alabama	7232	8.2 (8.0, 8.4)	8.9	14.3	17.6	17.0	2000–2008	8.1^a^ (4.9, 11.4)	< 0.001	2008–2017	0.4 (-1.5, 2.4)	0.628	4.0^a^ (2.4, 5.6)	< 0.05
Alaska	1364	11.1 (10.5,11.7)		17.0	17.7	18.9	2005–2017	0.7 (-2.1, 3.6)	0.589				0.7 (-2.1, 3.6)	> 0.05
Arizona	14,736	13.1 (12.9,13.3)	10.3	24.9	22.6	18.1	2000–2005	16.5^a^ (11.1, 22.1)	< 0.001	2005–2010	4.3 (-0.2, 9.0)	0.059	3.7^a^ (1.4, 6.0)	< 0.05
							2010–2015	-2.6 (-6.4, 1.3)	0.153	2015–2017	-10.6 (-21.2, 1.4)	0.073		
California	71,925	10.8 (10.8,10.9)	11.4	20.3	23.3	21.3	2000–2008	7.6^a^ (6.4, 8.9)	< 0.001	2008–2015	2.4^a^ (1.1, 3.7)	0.002	3.9^a^ (2.8, 4.9)	< 0.05
							2015–2017	-5.3 (-12.2, 2.1)	0.139					
Connecticut	10,859	16.1 (15.8,16.4)	13.6	30.6	27.5	23.0	2000–2007	12.1^a^ (9.2, 15.2)	< 0.001	2007–2014	0.6 (-1.9, 3.2)	0.601	3.7^a^ (1.9, 5.6)	< 0.05
							2014–2017	-7.3 (-14, 0)	0.050					
District of Columbia	1442	12.5 (11.9,13.2)	14.2	30.6	17.6	25.1	2000–2017	2.1 (-0.1, 4.3)	0.061				2.1 (-0.1, 4.3)	> 0.05
Florida	38,757	10.6 (10.5,10.7)	12.4	19.9	19.9	18.2	2000–2008	7.4^a^ (5.3, 9.6)	< 0.001	2008–2017	-0.3 (-1.7, 1.0)	0.602	3.2^a^ (2.1, 4.4)	< 0.05
Hawaii	3335	13.3 (12.8,13.7)	11.5	22.5	21	18.4	2000–2010	6.0^a^ (2.7, 9.3)	0.001	2010–2017	-3.1 (-7.2, 1.2)	0.140	2.1 (-0.2, 4.5)	> 0.05
Idaho	3772	14.1 (13.7,14.6)	8.8	25.0	25.1	19.2	2000–2008	13.4^a^ (7.8, 19.2)	< 0.001	2008–2017	-2.2 (-4.9, 0.7)	0.122	4.9^a^ (2.2, 7.6)	< 0.05
Indiana	12,196	10.4 (10.2,10.6)	11.2	18.8	19.5	17.0	2000–2010	6.3^a^ (4.9, 7.7)	< 0.001	2010–2017	-1.0 (-2.8, 0.9)	0.267	3.2^a^ (2.2, 4.3)	< 0.05
Kansas	7027	13.8 (13.5,14.1)	13.5	24.2	29.2	21.4	2000–2010	9.0^a^ (6.0, 12.1)	< 0.001	2010–2017	-3.3 (-6.7, 0.2)	0.127	3.7^a^ (1.7, 5.8)	< 0.05
Kentucky	10,053	12.5 (12.2,12.7)	9.5	24.3	26.5	24.7	2000–2009	10.8^a^ (8.4, 13.2)	< 0.001	2009–2017	1.0 (-0.8, 2.8)	0.246	6.1^a^ (4.7–7.4)	< 0.05
Louisiana	8926	10.7 (10.5,11.0)	9.7	19.8	26.1	24.3	2000–2013	7.7^a^ (5.9, 9.5)	< 0.001	2013–2017	-2.3 (-9.0, 4.9)	0.495	5.2^a^ (3.3, 7.3)	< 0.05
Maine	3238	12.5 (12.1,12.9)	6.9	18.1	28.8	18.8	2000–2015	8.1^a^ (5.8, 10.5)	< 0.001	2015–2017	-19.8 (-45.4,17.8)	0.237	4.4 (-0.2, 9.2)	> 0.05
Massachusetts	21,047	16.8 (16.6,17.1)	13.3	33.2	32.7	26.7	2000–2007	16.1^a^ (12.1, 20.2)	< 0.001	2007–2017	-1.0 (-2.4, 0.4)	0.157	5.7^a^ (4.2, 7.3)	< 0.05
Minnesota	10,618	11.0 (10.8,11.2)	9.6	17.3	20.9	17.5	2000–2013	5.9^a^ (4.4, 7.4)	< 0.001	2013–2017	-4.2 (-10.3, 2.3)	0.181	3.4^a^ (1.7, 5.2)	< 0.05
Mississippi	4287	9.5 (9.2, 9.8)		17.7	20.1	14.8	2003–2011	7.2^a^ (3.1, 11.4)	0.002	2011–2017	-4.1 (-8.9, 1.0)	0.102	2.2 (-0.6, 5.0)	> 0.05
Missouri	11,997	10.9 (10.7,11.1)	11.3	22.5	19.2	19.3	2000–2008	9.7^a^ (7.3, 12.1)	< 0.001	2008–2017	-1.2 (-2.6, 0.1)	0.071	3.8^a^ (2.6, 5.0)	< 0.05
Montana	2370	13.0 (12.4,13.5)	10.7	24.7	24.2	14.9	2000–2015	4.4^a^ (2.6, 6.2)	< 0.001	2015–2017	-24.0 (-46.3, 7.6)	0.112	0.6 (-3.3, 4.7)	> 0.05
Nebraska	4079	12.5 (12.1,12.9)	9.9	24.7	24.2	23.7	2000–2008	9.4^a^ (5.1, 13.8)	< 0.001	2008–2017	0.3 (-2.1, 2.7)	0.807	4.5^a^ (2.4, 6.6)	< 0.05
New Hampshire	3252	12.8 (12.4,13.3)	10.5	22.1	20.7	20.6	2000–2006	18.1^a^ (6.9, 30.5)	0.003	2006–2017	-0.3 (-2.9, 2.4)	0.815	5.9^a^ (2.2, 9.7)	< 0.05
New Jersey	26,846	16.1 (16.0,16.3)	16.7	32.0	29.2	28.2	2000–2010	7.0^a^ (5.7, 8.2)	< 0.001	2010–2017	-1.4 (-2.9, 0.2)	0.086	3.5^a^ (2.6, 4.4)	< 0.05
Ohio	25,253	11.7 (11.5,11.8)	9.9	21.4	22.3	23.6	2000–2009	9.6^a^ (7.3, 12.1)	< 0.001	2009–2017	1.1 (-0.7, 2.9)	0. 217	5.5^a^ (4.2, 6.9)	< 0.05
Oregon	7933	11.2 (10.9,11.4)	9.5	17.9	20.8	18.5	2000–2008	11.1^a^ (6.3, 16.3)	< 0.001	2008–2017	0.7 (-1.9, 3.4)	0.583	5.5^a^ (3.1, 7.9)	< 0.05
Tennessee	13,141	11.2 (11.0,11.4)	10.1	19.8	20.5	19.1	2000–2008	11.3^a^ (7.6, 15.1)	< 0.001	2008–2017	-1.1 (-3.1, 0.9	0.240	4.5^a^ (2.7, 6.4)	< 0.05
Virginia	15,605	10.6 (10.4,10.7)	10.4	20.0	21.2	18.9	2000–2009	10.4^a^ (7.6, 13.2)	< 0.001	2009–2017	-0.2 (-2.2, 1.8)	0.842	5.3^a^ (3.7, 6.9)	< 0.05
Washington	14,659	11.9 (11.7,12.1)	10.4	21.6	21.5	18.8	2000–2011	6.6^a^ (4.6, 8.7)	< 0.001	2011–2017	-2.9 (-6.3, 0.6)	0.093	3.1^a^ (1.5, 4.8)	< 0.05
West Virginia	4593	12.7 (12.3,13.1)	10.3	18.6	23.0	25.7	2000–2012	7.2^a^ (5.4, 9.1)	< 0.001	2012–2017	-1.6 (-6.3, 3.4)	0.495	4.6^a^ (2.8, 6.4)	< 0.05
Wyoming	1272	12.7 (12.0,13.5)		14.7	17.8	24.5	2003–2017	-0.7 (-3.1, 1.7)	0.536				-0.7 (-3.1, 1.7)	> 0.05
**3. Decreasing trends**														
Colorado	11,810	13.0 (12.8,13.2)	10.2	25.4	24.1	21.1	2000–2010	9.1^a^ (7.8, 10.4)	< 0.001	2010–2017	-3.6^a^ (-4.9, -2.2)	< 0.001	3.7^a^ (2.8, 4.6)	< 0.05
Illinois	28,879	12.3 (12.2,12.5)	12.3	22.0	26.5	22.5	2000–2007	8.9^a^ (7.1, 10.8)	< 0.001	2007–2015	2.8* (1.5, 4.1)	0.001	3.9^a^ (2.6, 5.1)	< 0.05
							2015–2017	-8.5^a^ (-16.2, -0.1)	0.049					
Maryland	12,866	12.8 (12.6,13.1)	15.7	27.2	23.6	19.7	2000–2013	4.7^a^ (3.3, 6.0)	< 0.001	2013–2017	-8.5^a^ (-14.4, -2.3)	0.012	1.4 (-0.3, 3.1)	> 0.05
Michigan	20,624	11.1 (10.9,11.2)	11.5	20.8	19.4	17.4	2000–2010	7.0^a^ (5.3, 8.7)	0.001	2010–2017	-2.5^a^ (-4.7, -0.4)	0.025	3.0^a^ (1.8, 4.2)	< 0.05
Nevada	6536	13.6 (13.3,13.9)	12.1	28.7	27.5	21.6	2000–2013	6.0^a^ (3.9, 8.1)	< 0.001	2013–2017	-8.8^a^ (-16.4,-0.6)	0.038	2.3 (0, 4.7)	> 0.05
New York	57,216	15.7 (15.5,15.8)	14.0	30.0	30.2	27.2	2000–2003	4.4 (-0.4, 9.3)	0.068	2003–2008	12.1^a^ (9.6, 14.8)	< 0.001	3.9^a^ (2.8, 5.1)	< 0.05
							2008–2013	2.6^a^ (0.7, 4.6)	0.015	2013–2017	-4.2^a^ (-5.9, -2.4)	0.001		
North Carolina	18,409	10.6 (10.5,10.8)	9.4	21.6	20.0	17.4	2000–2003	2.0 (-14.7, 22.1)	0.798	2003–2006	26.8 (-5.3, 69.8)	0.096	3.8 (-1.6, 9.5)	> 0.05
							2006–2013	2.4 (-1.3, 6.3)	0.172	2013–2017	-7.4^a^ (-13.2,-1.2)	0.027		
Pennsylvania	41,361	17.1 (17.0,17.3)	14.9	33.2	29.2	26.5	2000–2010	8.2^a^ (7.1, 9.2)	< 0.001	2010–2017	-3.4^a^ (-4.7, -2.2)	< 0.001	3.2^a^ (2.5, 3.9)	< 0.05
Rhode Island	3220	16.1 (15.5,16.7)	12.2	32.5	26.5	24.3	2000–2010	10.8^a^ (7.5, 14.2)	< 0.001	2010–2017	-4.8^a^ (-8.6, -0.9)	0.020	4.1^a^ (1.8, 6.4)	< 0.05
South Carolina	8117	9.5 (9.3, 9.7)	10.1	17.8	21.0	17.0	2000–2012	7.7^a^ (6.6, 8.7)	< 0.001	2012–2017	-4.0^a^ (-6.6, -1.3)	0.007	4.1^a^ (3.1, 5.1)	< 0.05
Texas	46,306	10.8 (10.7,10.9)	11.7	23.3	20.9	19.2	2000–2006	10.1^a^ (7.3, 12.9)	< 0.001	2006–2011	3.2 (-0.3, 6.9)	0.070	3.2^a^ (1.9, 4.5)	< 0.05
							2011–2017	-3.3^a^ (-4.8, -1.8)	0.001					

^a^The difference was statistically significant

### Race/ethnicity and age differences in state-level of TC incidence

**Fig. 1:**
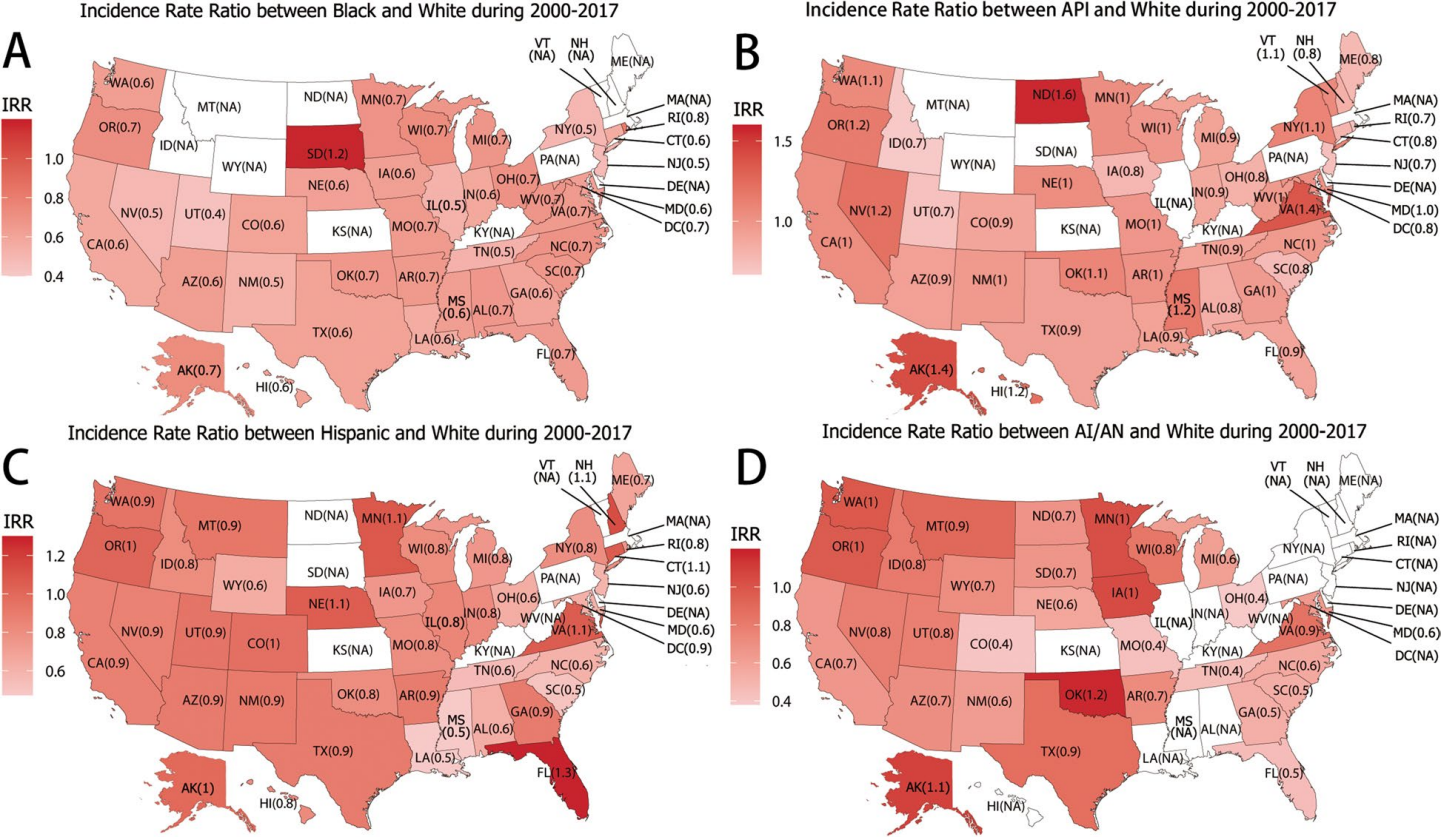
Incidence Rate Ratio between non-White and White during 2000–2017. **A**: Incidence Rate Ratio between Black and White during 2000–2017; **B**: Incidence Rate Ratio between API and White during 2000–2017; **C**: Incidence Rate Ratio between Hispanic and White during 2000–2017; **D**: Incidence Rate Ratio between AI/AN and White during 2000–2017

**Fig. 2:**
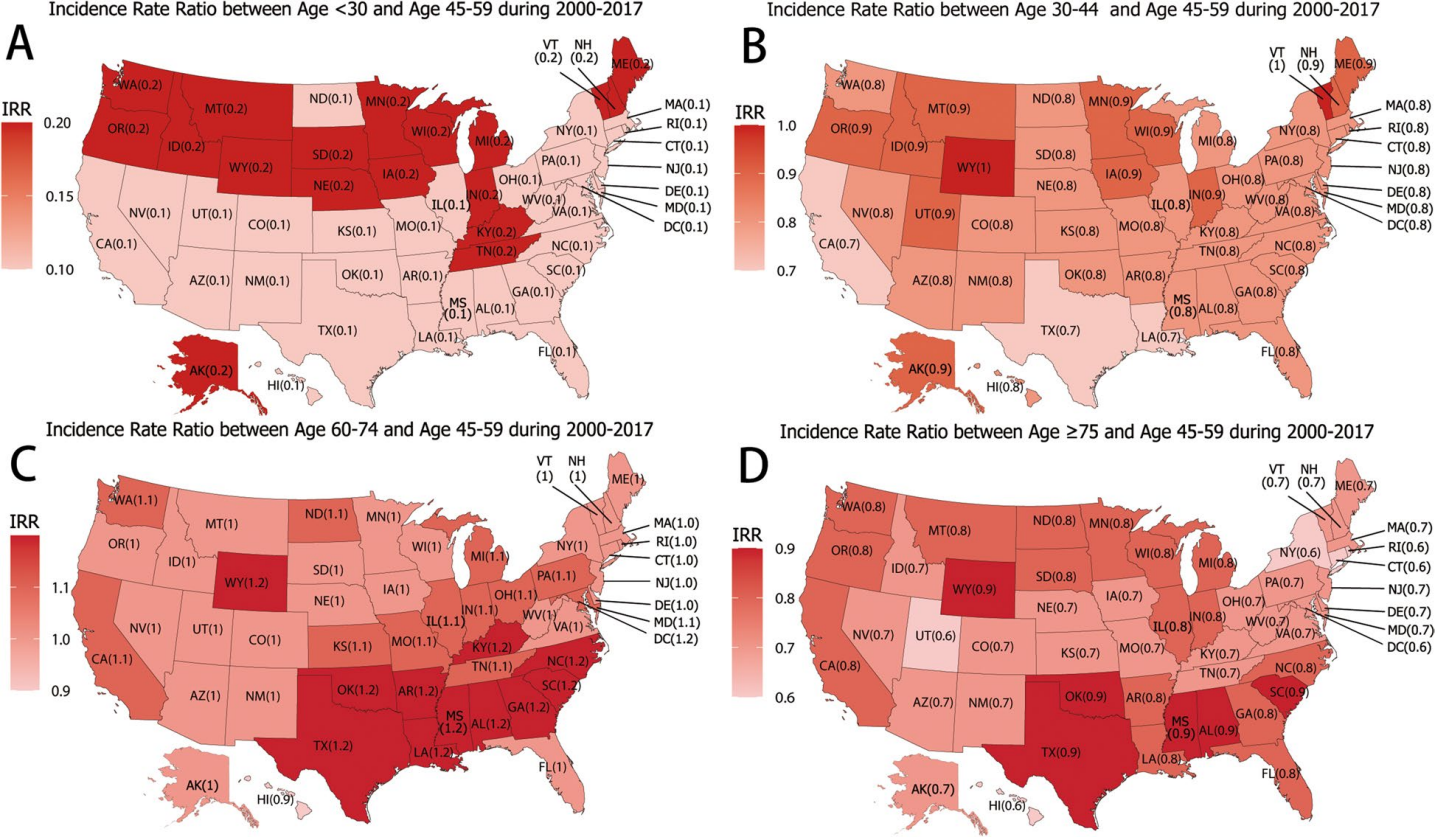
Incidence Rate Ratio between other age group and 45–59 age group during 2000–2017. **A**: Incidence Rate Ratio between < 30 age group and 45-59 age group during 2000-2017; **B**: Incidence Rate Ratio between 30-44 age group and 45-59 age group during 2000-2017; **C**: Incidence Rate Ratio between 60-74 age group and 45-59 age group during 2000-2017; **D**: Incidence Rate Ratio between ≥ 75 age group and 45-59 age group during 2000-2017.

We analyzed the incidence rate ratio (IRR) at the state level by race/ethnicity (including Blacks/Whites, APIs/Whites, AIs/ANs/Whites, and Hispanics/Whites). We found that the lowest IRRs were concentrated in the central region and the highest IRRs were scattered across the USA. As shown in Fig. 1, Blacks had the highest IRR in South Dakota (IRR, 1.2) and the lowest in Utah (IRR, 0.4); APIs had the highest IRR in North Dakota (IRR, 1.6) and the lowest in Idaho (IRR, 0.7); AIs/ANs had the highest IRR in Oklahoma (IRR, 1.2) and the lowest in Ohio (IRR, 0.4); Hispanics had the highest IRR in Florida (IRR, 1.3) and the lowest in Louisiana(IRR, 0.5). We also found that the highest IRRs between the < 30 age group and 45–59 age groups were distributed in the northern United States(Fig. 2A); and so was the distribution of the 30–44 age group (Fig. 2B). On the other hand, the highest IRRs between the 60–75 and 45–59 age group were distributed in the southern United States (Fig. 2C), and so was the distribution of the > 75 age group (Fig. 2D).

### The ecological correlation between state-level obesity/physical activity and TC incidence trends

Obesity of the state-level increased gradually from 2011(mean, 27.6%; range, 20.7%–34.9%) to 2017(mean, 30.7%; range, 22.6%-38.1%). During 2011–2017, Mississippi (35.8%) had the highest prevalence of obesity and Colorado (21.3%) had the lowest prevalence. Blacks (36.6%) had the highest prevalence of obesity, followed by Hispanics (31.1%), and Whites (27.4%).

State-level physical activity level remained stable between 2011(mean, 50.9%; range, 33.8%–61.8%) and 2017 (mean, 49.9%; range, 19.6%–59.7%). During 2011–2017, Oregon (60.6%) had the highest level of physical activity and Mississippi (40.0%) had the lowest level. Whites (36.6%) had the highest level of physical activity, followed by Blacks (31.1%), and Hispanics (27.4%).

**Fig. 3:**
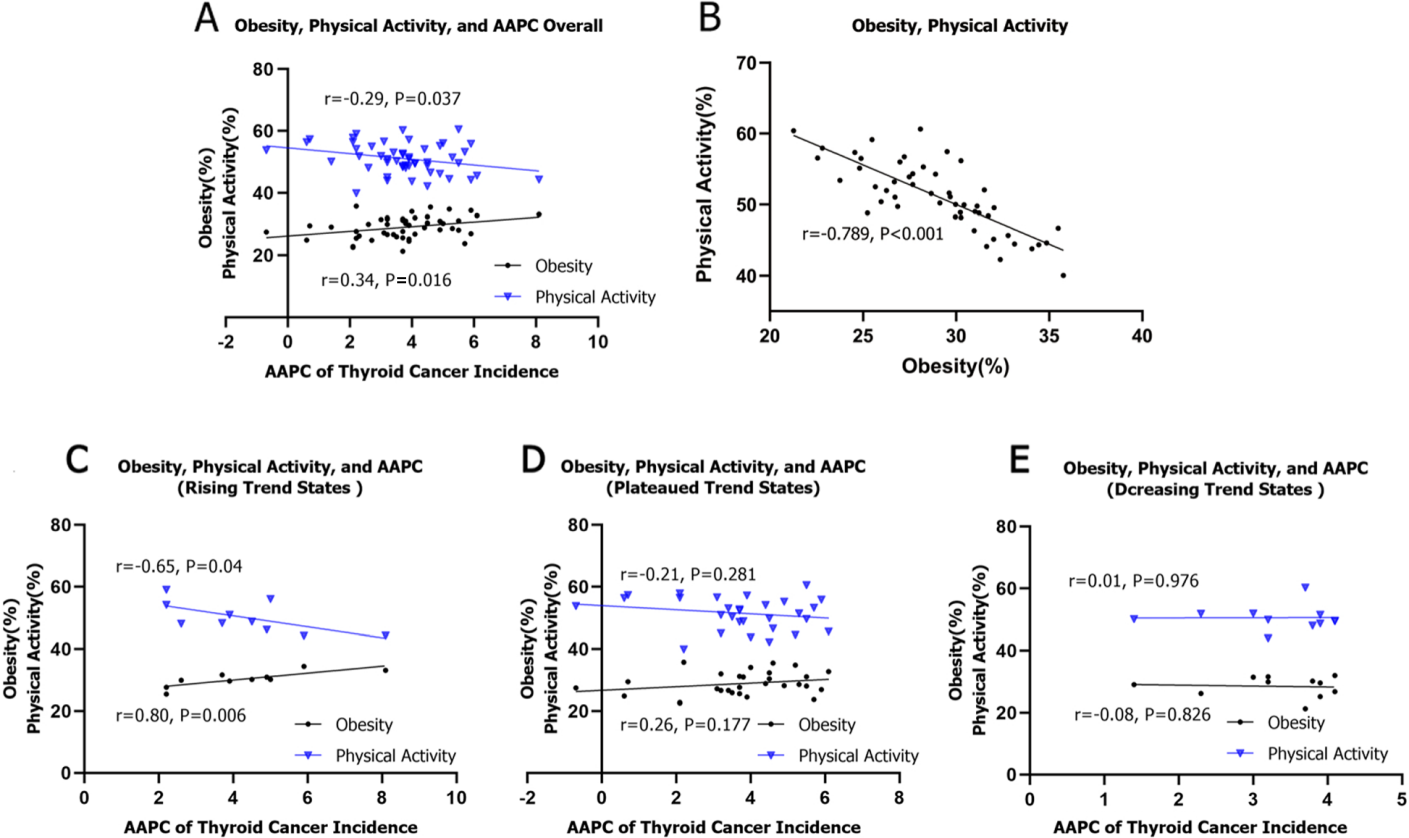
The correlation between obesity and physical activity level, thyroid cancer AAPC. The abscissa represents state-level the average obesity prevalence during 2011–2017; the ordinate represents the average physical activity ratio at the state-level; the Black dots and blue triangle represent states. **A**: Correlation between the AAPC of the thyroid cancer and physical activity level, obesity; **B**: Correlation between physical activity level and obesity; **C**: Correlation between AAPC of the Thyroid Cancer and physical activity level, obesity in rising trend states; **D**: Correlation between AAPC of the Thyroid Cancer and physical activity level, obesity in plateaued trend states; **E**: Correlation between AAPC of the Thyroid Cancer and physical activity level, obesity in decreasing trend states

National TC incidence trend and obesity prevalence at state-level showed a weak correlation (*r* = *0.34, P* = *0.016*); and physical activity level showed a weak correlation (*r* = *-0.29, P* = *0.037*) (Fig. 3A). We found a strong, inverse correlation between physical activity level and obesity prevalence(*r* = *-0.79, P* < *0.001*) (Fig. 3B). Rising trend states had an extremely strong correlation between AAPC and obesity prevalence (*r* = *0.80, p* = *0.006*), and a strong correlation with physical activity level(*r* = *-0.65, P* = *0.04*) (Fig. 3C). There was no correlation between AAPC of TC incidence and obesity prevalence/physical activity level within states where AAPC were either with decreasing or plateauing trend (Fig. 3D, Fig. 3E). Subgroup correlation analysis showed that state-level AAPC was moderately correlated with the prevalence of obesity in Whites (*r* = *0.49, P* = *0.001*), and weakly correlated with physical activity level (*r* = *-0.34, P* = *0.02*). There was no correlation between state-level AAPC and obesity prevalence/physical activity level in Blacks (obesity prevalence: *r* = *-0.12, P* = *0.590*; physical activity level: *r* = *-0.4, P* = *0.054*), Hispanics (obesity prevalence: *r* = *-0.21, P* = *0.242*; physical activity level: *r* = *0.27, P* = *0.131*), males (obesity prevalence: *r* = *0.25, P* = *0.079*; physical activity level: *r* = *-0.15, P* = *0.280*), females (obesity prevalence: *r* = *0.19, P* = *0.187*; physical activity level: *r* = *-0.17, P* = *0.246*) (Supplementary Figure. S1 and Supplementary Figure. S2).

## Discussion

In this study, a significant reduction in TC incidence was observed in the USA since 2015. However, the incidence rate continued to increase in 10 states, kept plateaued in 29 states and decreased in 11 states in recent years. We reported a striking state-level disparity between Whites and other racial/ethnic groups, as well as between the 45–59 age group and other age groups. Finally, a positive correlation between state-level obesity and TC incidence trend, and an inverse correlation between state-level physical activity and TC incidence trend were observed.

Similar to previous studies, the female incidence rate was still much higher than male, Whites and APIs incidence rate was the highest and Blacks were the lowest [5]. Similar to recent reports [28], we observed the highest incidence of TC was in the 60–74 age group. The incidence trends were divided into three segments during the study period. Incidence rate of TC increased rapidly during 2000–2009, and overdiagnosis was believed to be a major contribution. The incidence trends continued to increase [5, 6], but at a slower rate during 2009–2015, then decreased during 2015–2017. The trend variation during 2000–2017 may be related to the guidelines released by the American Thyroid Association in 2009 and 2015 [6]. When stratified by sex and race/ethnicity, the TC incidence trends of male, AIs/ANs, and Hispanics were not observed a significant decline. Previous studies have found that the TC incidence in male was more associated with obesity than in female [29], and our ecological association study support this view. During 2000–2017, the AIs/ANs had the maximum AAPC among all race/ethnicity, and the latest APC had not declined significantly in recent years. The cold climate in Alaska may be related to thyroid cancer [22]. Besides, a high prevalence of metabolic syndrome in the AIs/ANs population had been proposed [30], and metabolic syndrome was suspected to be associated with TC [31].

During 2000–2017, the TC incidence in almost all states was increasing. The largest increase was observed during 2000–2009 and began to decline after 2015. But Joinpoint regression analysis showed that incidence rates continued to rise in 10 states, including Arkansas, Delaware, Georgia, Iowa, New Mexico, North Dakota, Oklahoma, South Dakota, Vermont, and Wisconsin. Moreover, TC incidence in 29 states retain plateaued. It is well known that the burden of obesity and metabolic syndrome are highly variable regionally in the USA [30]. Our ecological correlation analysis showed that state-level AAPC had a strong correlation with the obesity prevalence in the states where the incidence trend continued to rise, and a strong inverse correlation between the state-level AAPC and physical activity level was also observed. High obesity prevalence in these states may be an important factor for the continuously increasing TC incidence. In addition, environmental factors may also have an impact on the diversities of the TC incidence trend among states. In the middle of the last century, the USA conducted a large number of atmospheric nuclear tests in Nevada and New Mexico. Radioactive dust generated by nuclear tests, such as radioactive iodine, may blown to pastures in the Midwest, and polluted local water sources [19], and the ionizing radiation was the best-known exposure factor in TC. In addition, due to a large number of applications in chemical pesticides, the environmental pollution is increasing, including nitrate pollution in water. A study conducted in Iowa suggests that the risk of TC is positively correlated with nitrate > 5 ppm in the public water supply system [32]. Similar report was also discovered in Wisconsin [33]. A study from North Dakota has also shown that the increased incidence of TC may also be related to urban water use [19]. We found that most of the states with a continuous increasing TC incidence were located in the Midwest regions. Flourishing agricultural development and the use of pesticides and fertilizers in these areas may lead to environmental pollution, and then nitrate and other careinogens may be absorbed into the human body. The economic development in the Midwest regions is backward. Passage of the Affordable Care Act in 2010, which could help people in the Midwest more access to health care as revenge. This factor may also partly explain the geographic disparity of TC incidence in the USA.

Although many studies have explored racial/ethnic and age disparity in TC incidence [10, 11], state-level disparity have not been studied. In our study, the TC incidence of white among states was similar, other race/ethnicity had significant variations in TC incidence among states. Blacks morbidity in TC was usually considered the lowest. Our study found that Blacks incidence of TC in South Dakota was the highest, which was 1.2-times higher than local Whites. However, the TC incidence of Blacks in other states was lower than local Whites. Hispanics had the highest IRR (1.3) and the lowest IRR (0.5) in the southeast. The IRRs of AIs/ANs (0.4–1.2) and APIs (0.7–1.6) were dispersed among states. It has already been pointed out that Variations in TC risk according to racial/ethnic group and geographical residence may reflected socioeconomic or local environmental influences [34]. Several f associated factors such as goitrogenic exposure, diet, body size, and menstrual and reproductive events were also proposed [35]. We found that state-level racial/ethnic incidence of TC varied greatly in the USA. The causes for the variations remain unclear, it can be attriuted to environmental, socioeconomic and lifestyle factors. Studying the state-level racial/ethnic incidence may be helpful for health care sector formulate management policies. Local public health officials should explore the causes of disparity in-depth and formulate management plans. The young population had a high TC incidence in the northern USA, while the elderly had a high TC incidence in the south. The interesting observation could be partly explained by variation in metabolic burden. Metabolic syndrome was considered to be a risk factor for TC [36, 37], which was common in the elderly [38]. The high prevalence of metabolic syndrome and obesity in the southern United States [39], which may be an explanation for the state-level age disparity.

The causal relationship between TC and obesity had been proposed [17, 18]. Although the detailed link is not clear, mechanisms including hyperinsulinemia, chronic inflammation and changes in circulating fat factors (including leptin and adiponectin) had been proposed [18]. In the United States, the spatial distribution of the obesity rate was uneven. Islami found that the risk of overweight leading to cancer was the highest in the Southern and the Midwest, Alaska and Colombia of the USA [40]. Geographical variations in obesity prevalence may directly result in a state-level disparity in TC incidence. Long-term physical activity is an effective intervention to reduce adipose tissue and correct metabolic abnormalities, which may reduce cancer risk by lowering systemic pro-inflammatory biomarkers [18]. Intervention on metabolic factors may help reduce the incidence of TC, but it is difficult to directly achieve at the population-level. As individual behavioural choices tend to occur in a community context, reducing the prevalence of obesity will require primary prevention interventions at the individual and community levels to promote a healthy diet and physical activity [40].

There are two deficiencies in this study. Firstly, USCS does not provide detailed clinical information such as pathological type, such as clinical stage, tumor size, radiation exposure history, past personal history and BMI. Secondly, our ecological correlation analysis has inherent deficiencies, but ecological research is usually considered as a common method to evaluate the effect of macroscopic sanitary precautions [41].

In the USA, the TC incidence in 10 states was still increasing. In addition, state-level race/ethnicity and age incidence of TC varied considerably, and the causes remain unknown. Metabolic factors and environmental factors may be an explanation. Community and individuals need to pay attention to cancer-related problems caused by obesity prevalence and environmental pollution.

## Supplementary Information

**Additional file 1: Supplementary Figure S1.** The correlation between obesity and physical activitylevel, thyroid cancer AAPC by sex. The abscissa represents state-level the averageobesity prevalence during 2011-2017; the ordinatere presents the average physicalactivity ratio at the state-level ; the Black dots and blue triangle represent states.(A): Correlation between AAPC of the TC and physical activity level, obesity inmale; (B): Correlation between AAPC of the TC and physical activity level, obesityin female.

**Additional file 2: Supplementary Figure S2. **The correlation between obesity and physical activity level,thyroid cancer AAPC by race/ ethnicity. The abscissa represents state-level theaverage obesity prevalence during 2011-2017; the ordinatere presents the averagephysical activity ratio at the state-level; the Black dots and blue trianglerepresentstates.(A): Correlation between AAPC of the TC and physical activity level,obesity in White;(B): Correlation between AAPC of the TC and physical activity level,obesity in Black; (C): Correlationbetween AAPC of the TC and physical activity level,obesity in Hispanic.

## Data Availability

Anyone can visit and download needed data from the United State Cancer Statistics web site (https://wonder.cdc.gov/cancer-v2017.HTML). Besides, anyone can visit and download needed data from Behavioral Risk Factor Surveillance System web site ( https://nccd.cdc.gov/BRFSSPrevalence/rdPage.aspx?rdReport=DPH_BRFSS.ExploreByTopic&irbLocationType=StatesAndMMSA&islClass=CLASS14&islTopic=TOPIC09&islYear=2019&rdRnd=55326).
