# Supplementary material for: Thyroid cancer incidence trend and association with obesity, physical activity in the United States
Source: BMC Public Health. 2022 Jul 12;22:1333. doi: 10.1186/s12889-022-13727-3 (PMC9281136; doi:10.1186/s12889-022-13727-3)
Supplement: Supplementary file 1 — Additional file 1: Supplementary Figure S1. The correlation between obesity and physical activitylevel, thyroid cancer AAPC by sex. The abscissa represents state-level the averageobesity prevalence during 2011-2017; the ordinatere presents the average physicalactivity ratio at the state-level ; the Black dots and blue triangle represent states.(A): Correlation between AAPC of the TC and physical activity level, obesity inmale; (B): Correlation between AAPC of the TC and physical activity level, obesityin female. [file 12889_2022_13727_MOESM1_ESM.pdf]

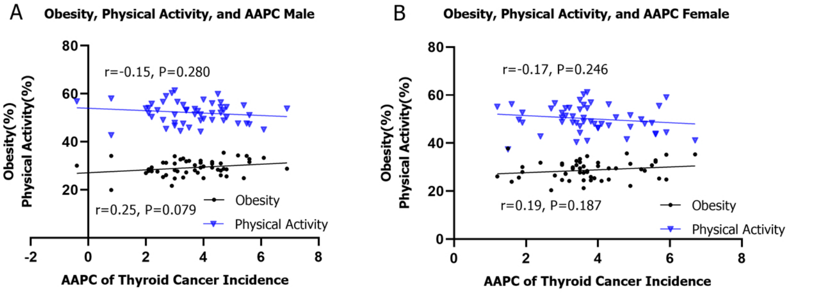

Supplementary Figure. S1: The correlation between obesity and physical activity level, thyroid cancer AAPC by sex. The abscissa represents state-level the average obesity prevalence during 2011–2017; the ordinate represents the average physical activity ratio at the state-level; the Black dots and blue triangle represent states.

(A): Correlation between AAPC of the TC and physical activity level, obesity in male;

(B): Correlation between AAPC of the TC and physical activity level, obesity in female.
