# Supplementary material for: Thyroid cancer incidence trend and association with obesity, physical activity in the United States
Source: BMC Public Health. 2022 Jul 12;22:1333. doi: 10.1186/s12889-022-13727-3 (PMC9281136; doi:10.1186/s12889-022-13727-3)
Supplement: Supplementary file 2 — Additional file 2: Supplementary Figure S2. The correlation between obesity and physical activity level,thyroid cancer AAPC by race/ ethnicity. The abscissa represents state-level theaverage obesity prevalence during 2011-2017; the ordinatere presents the averagephysical activity ratio at the state-level; the Black dots and blue trianglerepresentstates.(A): Correlation between AAPC of the TC and physical activity level,obesity in White;(B): Correlation between AAPC of the TC and physical activity level,obesity in Black; (C): Correlationbetween AAPC of the TC and physical activity level,obesity in Hispanic. [file 12889_2022_13727_MOESM2_ESM.pdf]

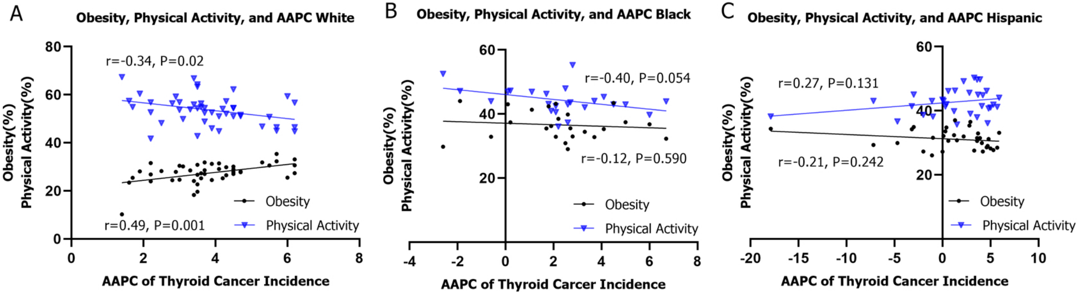

Supplementary Figure. S2: The correlation between obesity and physical activity level, thyroid cancer AAPC by race/ethnicity. The abscissa represents state–level the average obesity prevalence during 2011–2017; the ordinate represents the average physical activity ratio at the state–level; the Black dots and blue triangle represent states. (A): Correlation between AAPC of the TC and physical activity level, obesity in White; (B): Correlation between AAPC of the TC and physical activity level, obesity in Black; (C): Correlation between AAPC of the TC and physical activity level, obesity in Hispanic.
